# Explorando as Correlações entre as Manifestações Clínicas e Radiológicas Iniciais na Arterite de Takayasu

**DOI:** 10.36660/abc.20250534

**Published:** 2026-01-09

**Authors:** Anísio Uchoa Leite Santana, Samuel Katsuyuki Shinjo

**Affiliations:** 1 Faculdade de Medicina FMUSP Universidade de São Paulo São Paulo SP Brasil Faculdade de Medicina FMUSP, Universidade de São Paulo, São Paulo, SP – Brasil

**Keywords:** Arterite de Takayasu, Prognóstico, Vasculite Sistêmica

## Abstract

**Fundamento:**

As associações clínicas e de imagem iniciais na arterite de Takayasu (AT) são pouco definidas.

**Objetivos:**

Caracterizar as manifestações iniciais, suas associações e os resultados a longo prazo em uma coorte de pacientes com AT.

**Métodos:**

Um estudo de coorte retrospectivo unicêntrico (2000 a 2024) incluiu pacientes diagnosticados com AT. O nível de significância foi definido em p<0,05.

**Resultados:**

Dos 203 pacientes identificados, 54 foram excluídos devido a dados incompletos. A coorte final foi composta por 149 pacientes (89,9% do sexo feminino), com mediana de idade ao diagnóstico de 31 anos. Ao diagnóstico, 92,6% apresentavam sintomas. A claudicação dos membros superiores (36,2%) e inferiores (30,9%) foi frequente, juntamente com lesão vascular avançada, como estenose (85,9%) e oclusões (52,3%). A claudicação dos membros superiores foi predita independentemente pela redução dos pulsos nos membros superiores (OR=4,83; IC 95%=2,08-11,24; p=0,001) e pela oclusão da artéria subclávia direita (OR=8,06; IC 95%=1,94-33,44; p=0,004). A claudicação dos membros inferiores foi prevista pela oclusão da artéria subclávia direita (OR=6,65; IC 95%=2,05-21,61; p=0,002), espessamento da artéria subclávia direita (OR=5,12; IC 95%=1,18-22,71; p=0,029) e estenose da artéria subclávia esquerda (OR=2,71; IC 95%=1,21-60,56; p=0,016). Durante um acompanhamento mediano de 10 anos, apesar da remissão em 91,3% dos casos, houve aumento das comorbidades cardiovasculares e 26,8% dos pacientes necessitaram de cirurgia.

**Conclusões:**

A claudicação intermitente dos membros é um importante indicador prognóstico de lesão radiológica avançada e atraso no diagnóstico. Isso é reforçado pela dissociação a longo prazo entre alta remissão clínica e doença vascular progressiva, o que exige monitoramento vigilante.

## Graphical abstract

**Figura Central:**
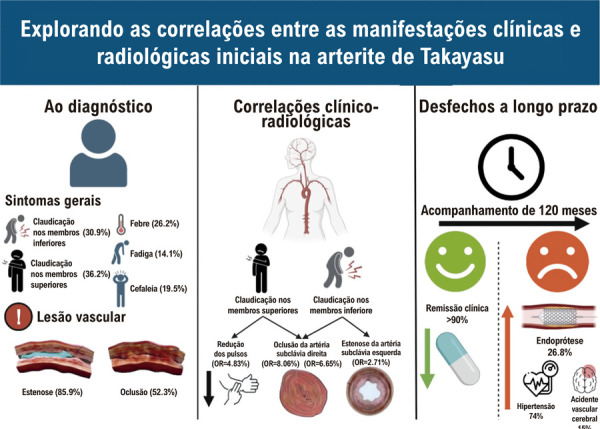
Explorando as Correlações entre as Manifestações Clínicas e Radiológicas Iniciais na Arterite de Takayasu

## Introdução

A arterite de Takayasu (AT) é uma forma rara de vasculite sistêmica primária que afeta predominantemente artérias de grande calibre, incluindo a aorta e seus principais ramos. A inflamação crônica desses vasos pode levar a estenoses, oclusões, ectasias ou aneurismas.^1^ Nesse contexto, o sistema de classificação proposto por Hata^2^ para AT facilita a caracterização anatômica das alterações vasculares e sua correlação com os sinais e sintomas clínicos observados em pacientes com AT.^3 , 4^

As manifestações clínicas mais comuns incluem febre, perda de peso, hipertensão arterial sistêmica, claudicação vascular dos membros, pressão arterial sistólica reduzida ou assimétrica entre os membros, vertigem e artralgia, entre outras.^5 , 6^ Os achados laboratoriais frequentemente revelam um aumento nos reagentes de fase aguda.^5 , 7^

No entanto, essas manifestações iniciais são heterogêneas, o que dificulta sua caracterização. Além disso, os estudos existentes na literatura não estabeleceram correlações consistentes entre as manifestações clínicas e os achados radiológicos iniciais,^6 , 8 - 20^ principalmente devido à ausência de uma análise temporal precisa que permitisse uma distinção clara entre manifestações precoces e tardias, bem como entre diferentes tipos de lesões vasculares. Quando avaliadas, essas alterações são frequentemente descritas de forma genérica, sem detalhes quanto ao tipo de lesão (estenose, oclusão, aneurisma etc.), o que limita a associação entre os achados clínicos e as alterações vasculares.

Portanto, o presente estudo teve como objetivo caracterizar as manifestações clínicas e radiológicas iniciais em uma coorte representativa de pacientes com AT e explorar as associações entre esses parâmetros. Além disso, o estudo avaliou a progressão da doença e o prognóstico dos pacientes.

## Métodos

### Desenho do estudo

Este estudo de coorte retrospectivo, unicêntrico, incluiu pacientes adultos diagnosticados com AT e tratados entre janeiro de 2000 e outubro de 2024 na Unidade de Vasculites de um hospital terciário. O estudo foi aprovado pelo Comitê de Ética em Pesquisa da instituição (CAAE 78848324.1.0000.0068).

Os critérios de inclusão exigiam que os participantes atendessem aos critérios de classificação de 1990 do *American College of Rheumatology* (ACR)^21^ e aos critérios de classificação de 2022 da *European Alliance of Associations for Rheumatology (EULAR)/ACR* ^22^ para AT.

Os critérios de exclusão envolveram a omissão de pacientes com dados clínicos ou radiológicos incompletos.

Este estudo foi relatado de acordo com a declaração *Strengthening the Reporting of Observational Studies in Epidemiology* (STROBE).^23^

### Coleta de dados

O estudo foi conduzido sistematicamente a partir de registros médicos eletrônicos, utilizando instrumentos padronizados e pré-parametrizados. Os dados coletados incluíram:

- Dados demográficos: sexo e etnia.
- Manifestações clínicas iniciais e dados laboratoriais: presença ou ausência de sintomas como perda de peso, febre, fadiga, cefaleia, tontura, distúrbios visuais, amaurose, artralgia, carotidínia, dor lombar, síncope, convulsões, acidente vascular cerebral, hipertensão arterial sistêmica, claudicação dos membros superiores e inferiores, parestesia, edema de membros, insuficiência aórtica, sopro cardíaco, angina, insuficiência cardíaca congestiva, infarto agudo do miocárdio, dispneia, dor abdominal, sopros (abdominais, aórticos, subclávios, carotídeos), eritema, pulsos reduzidos ou ausentes nos membros superiores, diferença de pressão arterial sistólica ≥ 20 mmHg entre os membros superiores e elevação de reagentes de fase aguda (proteína C-reativa - PCR e velocidade de hemossedimentação - VHS). Apenas os sintomas compatíveis com AT foram considerados, excluindo-se manifestações atribuíveis a outras etiologias (neoplásicas, infecciosas etc.).
- Resultados de imagem: Os exames de imagem ambulatoriais iniciais, incluindo arteriografia, angiotomografia computadorizada, angiorressonância magnética, ultrassonografia Doppler ou PET-CT de grandes vasos, foram avaliados quanto à presença de estenoses, oclusões, ectasias, aneurismas ou espessamento da parede nas seguintes artérias: coronária direita e esquerda, vertebral, carótida, subclávia, renal, mesentérica superior e inferior, tronco celíaco, ilíaca, femoral (superficial e profunda) e outras potencialmente afetadas.
- Tratamento: Foram administrados glicocorticoides, imunossupressores (IS), imunomoduladores (IM) e imunobiológicos.
- Evolução clínica: Foram documentadas complicações cirúrgicas e clínicas, bem como a mortalidade.

### Análise estatística

A avaliação da normalidade das variáveis foi realizada por meio do teste de Shapiro-Wilk. Os dados foram apresentados como média e desvio padrão, mediana e intervalo interquartil, ou frequências absolutas e relativas, conforme apropriado. Para a análise univariada, as comparações entre grupos independentes foram realizadas utilizando o teste t de Student não pareado para variáveis contínuas com distribuição normal e o teste U de Mann-Whitney para variáveis contínuas sem distribuição normal. As variáveis categóricas foram comparadas utilizando o teste qui-quadrado ou o teste exato de Fisher, dependendo da distribuição de frequência esperada, ou o teste de McNemar para dados categóricos pareados, dependendo do tipo de variável. Todas as variáveis que apresentaram diferença significativa nesta análise foram selecionadas para ajuste. As razões de chances (OR) ajustadas por sexo e idade, com intervalos de confiança (IC) de 95%, foram calculadas utilizando um modelo de regressão logística incondicional. O nível de significância foi definido em p < 0,05. O software estatístico SPSS versão 21.0 (Chicago, EUA) foi utilizado para as análises.

## Resultados

Entre 2000 e 2024, foi identificada uma coorte de 203 pacientes diagnosticados com AT. Destes, 54 pacientes foram excluídos devido a dados clínicos incompletos, 32 devido à falta de informações sobre os sintomas iniciais e manifestações clínicas ao diagnóstico e 22 devido a dados radiológicos vasculares insuficientes. Consequentemente, a análise final incluiu 149 pacientes.

A idade mediana ao diagnóstico foi de 31,0 anos (intervalo interquartil: 27,0-34,3 anos), e o intervalo mediano entre o início dos sintomas e o diagnóstico foi de 1,0 ano (intervalo interquartil: 0,0-2,3 anos), com 68,0% dos casos diagnosticados em até 12 meses após o início dos sintomas. Dos 149 pacientes, 80,5% eram do sexo feminino e 81,2% eram de etnia branca.

A Tabela 1 apresenta as manifestações clínicas iniciais ao diagnóstico. A maioria dos pacientes apresentou sintomas, predominantemente constitucionais, cefálicos, torácicos e musculoesqueléticos. Febre e perda de peso foram relatadas em aproximadamente 25% dos casos, seguidas por claudicação de membros superiores, dispneia e cefaleia. Manifestações menos frequentes incluíram alterações visuais, síncope, parestesia e sintomas abdominais ou cutâneos.

**Tabela 1:** – Manifestações clínicas no momento do diagnóstico em 149 pacientes com arterite de Takayasu

Parâmetros	n (%)
**Assintomático**	11 (7,4)
**Sintomático**	138 (92,6)
**Geral**	
Febre	39 (26,2)
Perda de peso	32 (21,5)
Fadiga	21 (14,1)
Mal-estar	21 (14,1)
**Cefálico**	
Dor de cabeça	29 (19,5)
Síncope	20 (13,4)
Tontura	18 (12,1)
Distúrbios visuais	16 (10,7)
Carotidínia	13 (8,7)
Convulsões	3 (2,0)
Amaurose	4 (2,7)
**Torácico**	
Dispneia	29 (19,5)
Angina	18 (12,1)
Dor no peito	2 (1,3)
**Membros**	
Claudicação do membro superior	54 (36,2)
Claudicação dos membros inferiores	46 (30,9)
Artralgia	24 (16,1)
Parestesia	9 (6,0)
Edema	5 (3,4)
**Outros**	
Eritema	12 (8,1)
Dor abdominal	9 (6,0)
Dor lombar	9 (6,0)

Os dados são expressos em frequência (%).

A Tabela 2 apresenta os achados clínicos e laboratoriais ao diagnóstico. Aproximadamente metade dos pacientes apresentou diminuição dos pulsos nos membros superiores; uma proporção semelhante apresentou diferença na pressão sistólica entre os membros superiores e hipertensão arterial sistêmica. Manifestações menos frequentes incluíram ausência completa de pulsos arteriais, insuficiência aórtica, acidente vascular cerebral e insuficiência cardíaca congestiva. Entre os sopros vasculares, os mais prevalentes foram os carotídeos, abdominais e cardíacos, seguidos pelos sopros subclávios e aórticos. Os marcadores de fase aguda estavam elevados em aproximadamente 40% dos pacientes.

**Tabela 2:** – Achados clínicos e laboratoriais no momento do diagnóstico em 149 pacientes com arterite de Takayasu

Parâmetros	n (%)
Pulsos reduzidos nos membros superiores	74 (49,7)
Diferença sistólica > 20 mmHg nos membros superiores	64 (43,0)
Hipertensão arterial sistêmica	64 (43,0)
Perda de pulso	21 (14,1)
Insuficiência aórtica	16 (10,7)
Acidente vascular cerebral	18 (12,1)
Insuficiência cardíaca congestiva	11 (7,4)
Infarto agudo do miocárdio	6 (4,0)
Outros	14 (9,4)
Sopros	
Carótida	32 (21,5)
Abdominal	21 (14,1)
Cardíaco	17 (11,4)
Subclávia	12 (8,1)
Aórtica	2 (1,3)
Reagentes de fase aguda elevados	56 (37,6)

Os dados são expressos em frequência (%).

A Tabela 3 detalha os achados angiográficos iniciais. A estenose foi a alteração mais frequente, seguida por oclusões, espessamento parietal e, em menor grau, ectasia ou aneurismas. As lesões predominaram nos vasos cervicais, particularmente nas artérias carótidas e subclávias, com predominância à esquerda. Na região torácica, se destacaram os aneurismas do arco aórtico e o espessamento da aorta torácica. Entre os vasos abdominais, as artérias renais foram as mais afetadas, seguidas por alterações na aorta abdominal e no tronco celíaco. Na região pélvica, as lesões mais comuns foram estenoses das artérias ilíacas.

**Tabela 3:** – Características angiográficas no momento do diagnóstico em 149 pacientes com arterite de Takayasu

	Estenose	Oclusão	Ectasia/Aneurisma	Espessamento
**Vasos afetados**	128 (85,9)	78 (52,3)	51 (34,2)	69 (46,3)
**Cervical**				
Carótida esquerda	49 (32,9)	24 (16,1)	4 (2,7)	31 (20,8)
Carótida direita	42 (28,2)	16 (10,7)	3 (2,0)	26 (17,4)
Vertebral esquerda	8 (5,4)	6 (4,0)	1 (0,7)	2 (1,3)
Vertebral direita	10 (6,7)	6 (4,0)	1 (0,7)	2 (1,3)
Braquicefálica	7 (4,7)	2 (1.3)	5 (3.4)	14 (9,4)
**Cardíaca**				
Coronária esquerda	3 (2,0)	0	0	0
Coronária direita	4 (2,7)	0	0	3 (2,0)
**Torácica**				
Arco aórtico	3 (2,0)	0	16 (10,7)	9 (6,0)
Aorta torácica	6 (4,0)	0	13 (8,7)	25 (16,8)
Subclávia esquerda	49 (32,9)	27 (18,1)	3 (2,0)	23 (15,4)
Subclávia direita	29 (19,5)	16 (10,7)	2 (1,3)	10 (6,7)
Axilar esquerda	3 (2.0)	1 (0,7)	0	0
Axilar direita	1 (0,7)	1 (0,7)	0	0
**Abdominal**				
Tronco celíaco	19 (12,8)	1 (0,7)	2 (1,3)	2 (1,3)
Aorta abdominal	14 (9,4)	5 (3.4)	9 (6,0)	26 (17,4)
Aorta infrarrenal	3 (2,0)	3 (2.0)	8 (5,4)	6 (4,0)
Renal esquerda	33 (22,1)	8 (5,4)	2 (1,3)	3 (2,0)
Renal direita	36 (24,2)	5 (3,4)	2 (1,3)	6 (4,0)
Mesentérica superior	15 (10,1)	12 (8,1)	1 (0,7)	6 (4,0)
Mesentérica inferior	6 (4,0)	3 (2,0)	0	0
**Pélvica**				
Ilíaca esquerda	10 (6,7)	4 (3.4)	5 (3.4)	3 (3,0)
Ilíaco direita	12 (8,1)	8 (5,4)	4 (2,7)	3 (2,0)
Femoral superficial	0	3 (2,0)	0	0
Femoral profundo	0	1 (0,7)	0	0
**Outros**	2 (1,3)	0	5 (3.4)	4 (2,7)

Os dados são expressos em frequência (%).

Além disso, realizamos uma análise abrangente da associação entre a presença de claudicação nos membros superiores ou inferiores e as apresentações clínicas e radiológicas iniciais detalhadas anteriormente nas Tabelas 1 , 2 e 3 . Os parâmetros que demonstraram associação estatística são apresentados na Tabela 4 . Para os membros superiores, foram identificadas correlações significativas com tontura, distúrbios visuais, diminuição dos pulsos nos membros superiores, hipertensão arterial sistêmica, estenose da artéria subclávia esquerda, oclusão da artéria subclávia direita e espessamento da artéria subclávia direita (todos com p<0,05). Em relação aos membros inferiores, foram observadas associações notáveis com sopro cardíaco, estenose da artéria subclávia esquerda, oclusão da artéria subclávia direita e espessamento da artéria subclávia direita (todos com p<0,05). Na análise multivariada, os preditores independentes para claudicação vascular do membro superior foram pulsos reduzidos no membro superior (OR=4,83; IC 95%=2,08-11,24; p<0,001) e oclusão da artéria subclávia direita (OR=8,06; IC 95%=1,94-33,44; p=0,004). Para claudicação do membro inferior, os principais preditores foram estenose da artéria subclávia esquerda (OR=2,71; IC 95%=1,21-60,56; p=0,016), oclusão da artéria subclávia direita (OR=6,65; IC 95%=2,05-21,61; p=0,002) e espessamento da artéria subclávia direita (OR=5,12; IC 95%=1,18-22,71; p=0,029).

**Tabela 4:** – Associações estatisticamente significativas foram encontradas entre a presença de claudicação nos membros superiores ou inferiores e as manifestações clínicas iniciais e achados radiológicos

Membros superiores	Claudicação vascular		Valor de p
	Presente (n=54)	Ausente (n=95)	
Tontura	11 (20,4)	7 (7,4)	0,034
Distúrbios visuais	10 (18,5)	6 (6,3)	0,028
Pulsos reduzidos nos membros superiores	42 (77,8)	32 (33,7)	<0,001
Hipertensão arterial sistêmica	16 (29,6)	48 (50,5)	0,016
Estenose da artéria subclávia esquerda	24 (44,4)	25 (26,3)	0,030
Oclusão da artéria subclávia direita	13 (24,1)	3 (3,2)	<0,001
Espessamento da artéria subclávia direita	8 (14,8)	2 (2,1)	0,005
**Membros inferiores**	**Presente (n=46)**	**Ausente (n=103)**	**Valor de p**
Sopro cardíaco	1 (2,2)	16 (15,5)	<0,001
Estenose da artéria subclávia esquerda	21 (45,7)	28 (27,2)	<0,001
Oclusão da artéria subclávia direita	11 (23,9)	5 (4,9)	0,001
Espessamento da artéria subclávia direita	7 (15,2)	3 (2,9)	0,010

Os dados são expressos em frequência (%).

As tabelas 5 e 6 apresentam um resumo dos dados de acompanhamento, tratamento e desfecho. O período mediano de acompanhamento foi de 120 (49-178) meses. Na avaliação final, mais de 90% dos pacientes estavam em remissão clínica. Houve uma redução significativa no uso de prednisolona (p<0,001) e de agentes IS/IM, como metotrexato (p<0,001), azatioprina (p<0,001) e leflunomida (p<0,001).

**Tabela 5:** – Tratamento e acompanhamento de pacientes com arterite de Takayasu

	Basal	Final	Valor de p
**Tempo de acompanhamento (meses)**	120 (49-178)		
**Tratamento**			
Prednisolona	107 (71,8)	28 (18,8)	<0,001
**Medicamentos IS/IM/IB**			
Nenhum	25 (16,8)	91 (61,1)	<0,001
Metotrexato	110 (73,8)	23 (15,4)	<0,001
Azatioprina	55 (36,9)	22 (14,8)	<0,001
Leflunomida	30 (20.1)	6 (4,0)	<0,001
Micofenolato de mofetila	26 (17,4)	8 (5,4)	<0,001
Infliximabe	25 (16,8)	15 (10.1)	0,009
Ciclofosfamida	12 (8,1)	1 (0,7)	0,003
Hidroxicloroquina	11 (7,4)	2 (1.3)	0,008
Tocilizumabe	11 (7,4)	4 (2,7)	0,023
Adalimumabe	4 (2,7)	1 (0,7)	0,248
Sulfassalazina	3 (2,0)	0	0,248
Abatacept	3 (2,0)	1 (0,7)	0,480
Etanercept	2 (1,3)	0	N / D
Rituximab	1 (0,7)	0	N / D
Colchicina	1 (0,7)	0	N / D
Certolizumabe pegol	1 (0,7)	0	N / D
Tacrolimus	0	0	N / D
Ácido acetilsalicílico	-	119 (79,9)	N / D
Estatinas	-	93 (62,4)	N / D

Os dados são expressos como mediana (percentis 25 a 75) ou frequência (%). Os valores de p foram calculados usando o teste de McNemar. Basal: no momento do diagnóstico de AT e início do acompanhamento ambulatorial; Final: última avaliação ambulatorial. IB: imunobiológico; IM: imunomodulador; IS: imunossupressor. NA (Não Aplicável): Teste estatístico não realizado devido à baixa frequência, dados faltantes ou porque a medicação foi avaliada apenas em um momento. O símbolo “–” indica dados não coletados sistematicamente no momento especificado, enquanto “0” indica que o parâmetro foi avaliado e o valor registrado foi zero.

**Tabela 6:** – Resultados clínicos, complicações e mortalidade durante o acompanhamento

	Basal	Final
**Doença em remissão**	-	136 (91,3)
**Complicações**		
AVC	18 (12,1)	23 (15,4)
Insuficiência cardíaca congestiva	11 (7,4)	22 (14,8)
Infarto agudo do miocárdio	6 (4,0)	12 (15,4)
Hipertensão arterial sistêmica	64 (43,0)	110 (73,8)
Insuficiência aórtica	16 (10,7)	25 (16,6)
TVP/EP	-	7 (4,7)
Neoplasias	-	5 (3.4)
Dislipidemia	-	84 (56,4)
Diabetes mellitus	-	18 (12.1)
**Vascular**		
Infarto/Isquemia de outros órgãos	-	6 (4,0)
Dissecção da aorta	-	5 (3.4)
**Cirurgias**		
Endoprótese	-	40 (26,8)
By-pass	-	9 (6,0)
Substituição da válvula aórtica	-	9 (6,0)
Morte	-	5 (3.4)

Os dados são expressos como mediana (25º - 75º percentil) ou frequência (%). Basal: no momento do diagnóstico de AT e início do acompanhamento ambulatorial; Final: última avaliação ambulatorial. AVC: acidente vascular cerebral; TVP: trombose venosa profunda; EP: embolia pulmonar. O símbolo “–” indica dados não aplicáveis ou não coletados sistematicamente no ponto de tempo especificado.

A taxa de mortalidade geral foi de 3,4%.

Durante o período de acompanhamento, se observou um aumento na prevalência de comorbidades, incluindo hipertensão arterial sistêmica, acidente vascular cerebral, insuficiência cardíaca congestiva e infarto agudo do miocárdio. Além disso, complicações vasculares como infarto de outros órgãos e dissecção da aorta também foram observadas ( Tabela 6 ).

Além disso, houve um aumento na frequência de procedimentos cirúrgicos, incluindo implante de endoprótese (26,8%), cirurgias de revascularização (6,0%) e substituições da válvula aórtica (6,0%). Os detalhes são apresentados na Tabela 1 (Suplemento) .

## Discussão

Nossos achados revelam que, no diagnóstico inicial de AT, 7,4% dos pacientes eram assintomáticos. Entre aqueles que apresentavam sintomas, as manifestações clínicas foram predominantemente inespecíficas. Aproximadamente metade dos pacientes demonstrou comprometimento vascular significativo, como pulsos diminuídos e discrepância na pressão arterial sistólica nos membros superiores. Os exames de imagem revelaram diversas alterações relacionadas a sequelas, incluindo estenoses e oclusões, potencialmente indicativas de diagnóstico tardio da doença. A claudicação dos membros superiores foi prevista pela redução dos pulsos nos membros superiores e pela oclusão da artéria subclávia direita, enquanto a claudicação dos membros inferiores não foi prevista por lesões nos vasos infrarrenais, mas sim pelo comprometimento grave das artérias subclávias.

Os pontos fortes deste estudo incluem uma amostra representativa de pacientes com AT, recrutados de acordo com os critérios de classificação EULAR/ACR de 2022.^22^ Embora este estudo seja retrospectivo, os dados foram coletados sistematicamente a partir de registros eletrônicos de saúde, utilizando instrumentos padronizados e pré-parametrizados, minimizando assim o viés de medição e garantindo a qualidade e uniformidade dos dados.

Notavelmente, diferentemente de outros estudos na literatura,^6 , 8 - 12 , 14 - 20^ nossa pesquisa avaliou especificamente as manifestações iniciais detalhadas e sua correlação com o prognóstico, considerando também os eventos cirúrgicos. Por exemplo, Alnabwani et al.^6^ se focaram exclusivamente em estenoses sem especificar o momento da aquisição das imagens, enquanto Gloor et al.^8^ identificaram os vasos afetados sem detalhar as alterações anatômicas. Da mesma forma, Cong et al.^9^ e Vanoli et al.^10^ ou não definiram claramente o período da aquisição das imagens ou não diferenciaram consistentemente entre os achados radiológicos iniciais e tardios.

Numerosos estudos^11 , 12 , 14 - 16^ não especificaram o momento das alterações radiológicas, o que impede uma correlação precisa com os sintomas iniciais. Mesmo estudos que consideraram achados precoces, como o de Karabacak et al.,^17^ não especificaram o tipo de anormalidades radiológicas detectadas. Além disso, as pesquisas conduzidas por Thakare et al.^18^ e Misra et al.,^19^ embora abordassem aspectos iniciais, limitaram suas análises prognósticas a comparações específicas — envolvimento renal ou estado do pulso, respectivamente — apresentando dados em formato de razão, o que restringe a correlação abrangente das características clínicas e radiológicas iniciais com o prognóstico geral. Notavelmente, diferentemente do nosso estudo, esses trabalhos não detalharam eventos cirúrgicos. Ademais, o estudo de Uchida et al.,^20^ apesar de fornecer dados iniciais e prognósticos, foi limitado por um curto período de acompanhamento de apenas dois anos e também omitiu relatos de intervenções cirúrgicas.

Nossos achados revelaram que, no momento do diagnóstico de AT, 92,6% dos pacientes apresentavam sintomas. No entanto, estes exibiam sintomas clínicos variados e altamente inespecíficos, como febre e perda de peso. Em contrapartida, sintomas típicos, como claudicação vascular dos membros superiores e inferiores, embora mais específicos, estavam presentes em menos da metade dos pacientes. A claudicação dos membros superiores foi associada exclusivamente à diminuição dos pulsos nos membros superiores e à oclusão da artéria subclávia direita, apesar de sua correlação inicial com parâmetros como tontura e distúrbios visuais. Este achado sugere que a claudicação dos membros superiores serve como um marcador clínico significativo de doença vascular avançada, correlacionando-se com indicadores de dano vascular estabelecido e ressaltando a importância do diagnóstico tardio da doença. Nossos resultados indicam ainda que a claudicação não reflete necessariamente as manifestações clínicas iniciais, mas sim alterações vasculares específicas, enfatizando a importância do exame físico e da avaliação radiológica na avaliação do paciente.

A claudicação dos membros inferiores foi inicialmente associada a sopro cardíaco e diversas alterações radiológicas; a análise multivariada não revelou associação com lesões dos vasos da aorta infrarrenal ou com os sintomas iniciais. Em vez disso, foi relacionada a lesões vasculares em territórios suprarrenais, com preditores independentes incluindo estenose da artéria subclávia esquerda, oclusão da artéria subclávia direita e espessamento da artéria subclávia direita. Essa correlação inesperada sugere que a claudicação dos membros inferiores pode não refletir um envolvimento arterial localizado, mas sim indicar um comprometimento grave da artéria subclávia como marcador do estágio da doença, refletindo um comprometimento vascular significativo com repercussões hemodinâmicas globais. Assim como na claudicação dos membros superiores, esses achados corroboram a hipótese de um diagnóstico tardio, que ocorre quando o dano vascular já é funcionalmente significativo, ressaltando a importância dos exames de imagem no manejo do paciente.

Além disso, a prevalência de manifestações hemodinâmicas em aproximadamente metade dos pacientes no momento do diagnóstico, juntamente com alterações vasculares como estenoses e oclusões na maioria dos casos, fornece evidências para o diagnóstico tardio da AT.

A análise estatística se concentrou nos sintomas de claudicação vascular devido à sua relevância clínica e prevalência em nossa coorte. Embora manifestações sistêmicas como febre, fadiga e cefaleia fossem prevalentes no momento do diagnóstico, sua natureza inespecífica limita a capacidade de estabelecer correlações diretas e robustas com achados radiológicos específicos. Em contrapartida, sintomas mais específicos foram raros, o que impossibilitou uma análise estatística com poder suficiente para gerar conclusões válidas. Consequentemente, a análise foi conduzida com base na claudicação dos membros superiores e inferiores para correlacionar as manifestações clínicas iniciais e os achados de imagem na AT.

Nossos resultados revelaram ainda que, embora a grande maioria dos pacientes tenha alcançado a remissão, houve um aumento notável nas comorbidades cardiovasculares e na necessidade de intervenções cirúrgicas complexas. Isso indica que a remissão clínica não equivale invariavelmente à estabilidade anatômica ou à reversão de danos vasculares já estabelecidos, evidenciando uma dissociação crítica entre o controle da atividade inflamatória e a progressão a longo prazo dos danos vasculares.

A gravidade e a progressão da doença são evidenciadas pela alta incidência de intervenções cirúrgicas, como o implante de endopróteses em mais de um quarto dos pacientes, além de cirurgias de revascularização e substituições da válvula aórtica. A ocorrência de complicações vasculares graves, como dissecção aórtica durante o acompanhamento, acentua ainda mais essa progressão, indicando que a doença pode avançar com eventos vasculares extremamente graves. Isso é comprovado pelas causas de óbito, que incluíram eventos como acidente vascular cerebral isquêmico extenso e choque séptico. Esses dados ressaltam que, mesmo com o controle da inflamação, o dano vascular acumulado continua resultando em morbidade significativa, exigindo monitoramento rigoroso e contínuo.

Este estudo apresenta algumas limitações metodológicas. Primeiro, seu desenho retrospectivo pode introduzir viés de informação e depende da precisão e completude dos registros médicos eletrônicos. Segundo, por se tratar de um estudo unicêntrico, os resultados podem não ser generalizáveis para populações mais amplas com diferentes origens étnicas ou genéticas. Essas limitações ressaltam a necessidade de futuros estudos prospectivos multicêntricos para validar e ampliar nossos resultados.

## Conclusões

Este estudo elucida que a AT é caracterizada por sintomas inespecíficos que coincidem com lesões vasculares avançadas, como estenoses e oclusões. A correlação direta entre manifestações clínicas específicas, como claudicação, e essas lesões ressalta a importância prognóstica dos achados iniciais. A longo prazo, apesar da alta taxa de remissão clínica, a doença apresentou progressão, marcada pelo aumento de comorbidades cardiovasculares e pela necessidade de intervenções cirúrgicas, preenchendo assim uma lacuna na literatura sobre a progressão da AT. Estudos prospectivos multicêntricos são necessários para validar esses achados.

## Material suplementar

Table 1 (Supplement).
