# Supplementary material for: Explorando as Correlações entre as Manifestações Clínicas e Radiológicas Iniciais na Arterite de Takayasu
Source: Arq Bras Cardiol. 2026 Jan 9;122(12):e20250534. [Article in Portuguese] doi: 10.36660/abc.20250534 (PMC12978377; doi:10.36660/abc.20250534)
Supplement: Table 1 (Supplement). [file 0066-782x-abc-122-12-e20250534-suppl01.pdf]

Table 1 (Supplement). Vascular complications, surgical procedures, and causes of death during follow-up in patients with Takayasu arteritis

Category	n (%)
Aortic dissection	5 (3.4)
Infarction/Ischemia of other organs	
Upper limb	2 (1.3)
Retinal ischemia	1 (0.7)
Ischemic hepatitis	1 (0.7)
Mesenteric ischemia	1 (0.7)
Ischemia of right toe	1 (0.7)
Vascular procedures	
Endoprosthesis	40 (26.8)
Vascular bypass	9 (6.0)
Aortic valve replacement	9 (6.0)
Thoracic aortic aneurysm repair	2 (1.3)
Infrarenal aneurysm repair	1 (0.7)
Mitral valvuloplasty	1 (0.7)
Aortic valvuloplasty	1 (0.7)
Aortic coarctation repair	1 (0.7)
Aortic dissection repair	1 (0.7)
Renal angioplasty without stent	1 (0.7)
Percutaneous aortic angioplasty	1 (0.7)
Ascending aorta replacement + metallic valve	1 (0.7)
Pacemaker	1 (0.7)
Deaths	
No information	2 (1.3)
External cause	1 (0.7)
Extensive ischemic stroke	1 (0.7)
Septic shock	1 (0.7)

Data are expressed as frequency (%).
