# Supplementary material for: Explorando as Correlações entre as Manifestações Clínicas e Radiológicas Iniciais na Arterite de Takayasu
Source: Arq Bras Cardiol. 2026 Jan 9;122(12):e20250534. [Article in Portuguese] doi: 10.36660/abc.20250534 (PMC12978377; doi:10.36660/abc.20250534)
Supplement: Translation

# Translation: Exploring the Correlations between Initial Clinical and Radiological Manifestations in Takayasu’s Arteritis

**DOI:** 10.36660/abc.20250534i

**Authors:** Anísio Uchoa Leite Santana, Samuel Katsuyuki Shinjo

**Affiliations:** 1 Brazil Faculdade de Medicina FMUSP, Universidade de São Paulo, São Paulo, SP – Brazil

**Keywords:** Takayasu Arteritis, Prognosis, Systemic Vasculitis

## Abstract

**Background:**

Initial clinical and imaging associations in Takayasu’s arteritis (TAK) are poorly defined.

**Objectives:**

To characterize initial manifestations, their associations, and long-term outcomes in a TAK cohort.

**Methods:**

A single-center retrospective cohort study (2000 to 2024) included patients diagnosed with TAK. The significance level was set at p<0.05.

**Results:**

Among 203 patients identified, 54 were excluded due to incomplete data. The final cohort comprised 149 patients (89.9% female), with a median age at diagnosis of 31 years. At diagnosis, 92.6% were symptomatic. Claudication of upper (36.2%) and lower (30.9%) limbs was frequent, alongside advanced vascular damage like stenosis (85.9%) and occlusions (52.3%). Upper limb claudication was independently predicted by reduced upper limb pulses (OR=4.83; 95%CI=2.08-11.24; p=0.001) and right subclavian artery occlusion (OR=8.06; 95%CI=1.94-33.44; p=0.004). Lower limb claudication was predicted by right subclavian artery occlusion (OR=6.65; 95%CI=2.05-21.61; p=0.002), right subclavian artery thickening (OR=5.12; 95%CI=1.18-22.71; p=0.029), and left subclavian artery stenosis (OR=2.71; 95%CI=1.21-60.56; p=0.016). Over a median 10-year follow-up, despite 91.3% remission, cardiovascular comorbidities increased, and 26.8% required surgery.

**Conclusions:**

Limb claudication is a key prognostic indicator of advanced radiological damage and diagnostic delay. This is reinforced by a long-term dissociation between high clinical remission and progressive vascular disease, demanding vigilant monitoring.

## Introduction

Takayasu’s arteritis (TAK) is a rare form of primary systemic vasculitis that predominantly affects large-caliber arteries, including the aorta and its major branches. Chronic inflammation of these vessels can lead to stenosis, occlusions, ectasias, or aneurysms.^1^In this context, the classification system proposed by Hata^2^for TAK facilitates the anatomical characterization of vascular alterations and their correlation with the clinical signs and symptoms observed in patients with TAK.^3 , 4^

The most common clinical manifestations include fever, weight loss, systemic arterial hypertension, limb vascular claudication, reduced or asymmetric systolic blood pressure between limbs, vertigo, and arthralgia, among others.^5 , 6^Laboratory findings often reveal an increase in acute phase reactants.^5 , 7^

However, these initial manifestations are heterogeneous, complicating their characterization. Moreover, existing studies in the literature have not established consistent correlations between clinical manifestations and initial radiological findings,^6 , 8 - 20^primarily due to the absence of precise temporal analysis that would allow for a clear distinction between early and late manifestations, as well as between different types of vascular lesions. When evaluated, these changes are frequently described generically, lacking detail regarding the type of lesion (stenosis, occlusion, aneurysm, etc.), which limits the association between clinical findings and vascular alterations.

Therefore, the present study aimed to characterize the initial clinical and radiological manifestations in a representative cohort of patients with TAK and to explore the associations between these parameters. Additionally, the study evaluated the disease progression and prognosis of the patients.

## Methods

### Study design

This retrospective, single-center cohort study included adult patients diagnosed with TAK and treated between January 2000 and October 2024 at the Vasculitis Unit of a tertiary care hospital. The study received approval from the institution’s Research Ethics Committee (CAAE 78848324.1.0000.0068).

Inclusion criteria required participants to meet the 1990 American College of Rheumatology (ACR) classification criteria^21^and the 2022 European Alliance of Associations for Rheumatology (EULAR)/ACR classification criteria^22^for TAK.

Exclusion criteria involved the omission of patients with incomplete clinical or radiological data.

This study was reported in accordance with the Strengthening the Reporting of Observational Studies in Epidemiology (STROBE) statement.^23^

### Data collection

It was systematically conducted from electronic medical records using standardized and pre-parameterized instruments. The data collected included:

- Demographic data: sex and ethnicity.
- Initial clinical manifestations and laboratory data: presence or absence of symptoms such as weight loss, fever, fatigue, headache, dizziness, visual disturbances, amaurosis, arthralgia, carotidynia, lower back pain, syncope, seizures, stroke, systemic arterial hypertension, claudication of upper and lower limbs, paresthesia, limb edema, aortic insufficiency, heart murmur, angina, congestive heart failure, acute myocardial infarction, dyspnea, abdominal pain, bruits (abdominal, aortic, subclavian, carotid), erythema, reduced or absent upper limb pulses, systolic blood pressure difference ≥ 20 mmHg between upper limbs, and elevation of acute-phase reactants (C-reactive protein - CRP and erythrocyte sedimentation rate - ESR). Only symptoms consistent with TAK were considered, excluding manifestations attributable to other etiologies (neoplastic, infectious, etc.).
- Imaging findings: Initial outpatient imaging studies, including arteriography, computed tomography angiography, magnetic resonance angiography, Doppler ultrasound, or PET-CT of large vessels, were assessed for the presence of stenoses, occlusions, ectasias, aneurysms, or wall thickening in the following arteries: right and left coronary, vertebral, carotid, subclavian, renal, superior and inferior mesenteric, celiac trunk, iliac, femoral (superficial and deep), and others potentially affected.
- Treatment: Glucocorticoids, immunosuppressives (IS), immunomodulators (IM), and immunobiologicals (IB) were administered.
- Clinical course: Surgical and clinical complications, as well as mortality, were documented.

### Statistical analysis

It involved evaluating the normality of variables using the Shapiro-Wilk tests. Data were presented as mean and standard deviation, median and interquartile range, or absolute and relative frequencies, as appropriate. For the univariate analysis, comparisons between independent groups were performed using the unpaired Student’s t-test for normally distributed continuous variables, and the Mann-Whitney U test for non-normally distributed continuous variables. Categorical variables were compared using the chi-square test or Fisher’s exact test, depending on the expected frequency distribution, or McNemar’s test for paired categorical data were employed, depending on the variable type. All variables that differed significantly in this analysis were selected for adjustment. The sex-age-adjusted odds ratios (OR) with 95% confidence intervals (CI) were calculated using an unconditional logistic model. The significance level was set at p < 0.05. SPSS version 21.0 statistics software (Chicago, USA) was utilized for the analyses.

## Results

Between 2000 and 2024, a cohort of 203 patients diagnosed with TAK was identified. Of these, 54 patients were excluded due to incomplete clinical data, 32 due to missing information on initial symptoms and clinical manifestations at diagnosis, and 22 due to insufficient vascular radiological data. Consequently, the final analysis included 149 patients.

The median age at diagnosis was 31.0 years (interquartile range: 27.0-34.3 years), and the median interval between symptom onset and diagnosis was 1.0 year (interquartile range: 0.0-2.3 years), with 68.0% of cases diagnosed within 12 months of symptom onset. Among the 149 patients, 80.5% were female, and 81.2% were of white ethnicity.

Table 1 presents the initial clinical manifestations at diagnosis. The majority of patients exhibited symptoms, predominantly constitutional, cephalic, thoracic, and musculoskeletal. Fever and weight loss were reported in approximately 25% of cases, followed by upper limb claudication, dyspnea, and headache. Less frequent manifestations included visual changes, syncope, paresthesia, and abdominal or cutaneous symptoms.

**Table 1:** – Clinical manifestations at the time of diagnosis in 149 patients with Takayasu arteritis

Parameters	n (%)
**Asymptomatic**	11 (7.4)
**Symptomatic**	138 (92.6)
**General**	
Fever	39 (26.2)
Weight loss	32 (21.5)
Fatigue	21 (14.1)
Malaise	21 (14.1)
**Cephalic**	
Headache	29 (19.5)
Syncope	20 (13.4)
Dizziness	18 (12.1)
Visual disturbances	16 (10.7)
Carotidynia	13 (8.7)
Seizures	3 (2.0)
Amaurosis	4 (2.7)
**Thoracic**	
Dyspnea	29 (19.5)
Angina	18 (12.1)
Chest pain	2 (1.3)
**Limbs**	
Upper limb claudication	54 (36.2)
Lower limb claudication	46 (30.9)
Arthralgia	24 (16.1)
Paresthesia	9 (6.0)
Edema	5 (3.4)
**Others**	
Erythema	12 (8.1)
Abdominal pain	9 (6.0)
Low back pain	9 (6.0)

Data are expressed as frequency (%).

Table 2 presents the clinical and laboratory findings at diagnosis. Approximately half of the patients exhibited decreased pulses in the upper limbs; a similar proportion showed a difference in systolic pressure between the upper limbs and systemic arterial hypertension. Less frequent manifestations included complete absence of arterial pulses, aortic insufficiency, stroke, and congestive heart failure. Among vascular murmurs, the most prevalent were carotid, abdominal, and cardiac, followed by subclavian and aortic murmurs. Acute phase markers were elevated in approximately 40% of patients.

**Table 2:** – Clinical and laboratory findings at the time of diagnosis in 149 patients with Takayasu arteritis

Parameters	n (%)
Reduced upper limb pulses	74 (49.7)
Systolic difference > 20 mmHg in upper limbs	64 (43.0)
Systemic arterial hypertension	64 (43.0)
Pulse loss	21 (14.1)
Aortic insufficiency	16 (10.7)
Cerebrovascular accident	18 (12.1)
Congestive heart failure	11 (7.4)
Acute myocardial infarction	6 (4.0)
Others	14 (9.4)
Murmurs	
Carotid	32 (21.5)
Abdominal	21 (14.1)
Cardiac	17 (11.4)
Subclavian	12 (8.1)
Aortic	2 (1.3)
Elevated acute phase reactants	56 (37.6)

Data are expressed as frequency (%).

Table 3 details the initial angiographic findings. Stenosis was the most frequent alteration, followed by occlusions, parietal thickening, and, to a lesser extent, ectasis or aneurysms. Lesions predominated in the cervical vessels, particularly in the carotid and subclavian arteries, with a predominance on the left. In the thoracic region, aortic arch aneurysms and thickening of the thoracic aorta were notable. Among the abdominal vessels, the renal arteries were most affected, followed by alterations in the abdominal aorta and celiac trunk artery. In the pelvic region, the most common lesions were stenosis of the iliac arteries.

**Table 3:** – Angiographic characteristics at the time of diagnosis in 149 patients with Takayasu arteritis

	Stenosis	Occlusion	Ectasia/Aneurysm	Thickening
**Vessels Affected**	128 (85.9)	78 (52.3)	51 (34.2)	69 (46.3)
**Cervical**				
Left carotid	49 (32.9)	24 (16.1)	4 (2.7)	31 (20.8)
Right carotid	42 (28.2)	16 (10.7)	3 (2.0)	26 (17.4)
Left vertebral	8 (5.4)	6 (4.0)	1 (0.7)	2 (1.3)
Right vertebral	10 (6.7)	6 (4.0)	1 (0.7)	2 (1.3)
Brachycephalic	7 (4.7)	2 (1.3)	5 (3.4)	14 (9.4)
**Cardiac**				
Left coronary	3 (2.0)	0	0	0
Right coronary	4 (2.7)	0	0	3 (2.0)
**Thoracic**				
Aortic arch	3 (2.0)	0	16 (10.7)	9 (6.0)
Thoracic aorta	6 (4.0)	0	13 (8.7)	25 (16.8)
Left subclavian	49 (32.9)	27 (18.1)	3 (2.0)	23 (15.4)
Right subclavian	29 (19.5)	16 (10.7)	2 (1.3)	10 (6.7)
Left axillary	3 (2.0)	1 (0.7)	0	0
Right axillary	1 (0.7)	1 (0.7)	0	0
**Abdominal**				
Celiac trunk	19 (12.8)	1 (0.7)	2 (1.3)	2 (1.3)
Abdominal aorta	14 (9.4)	5 (3.4)	9 (6.0)	26 (17.4)
Infra-renal aorta	3 (2.0)	3 (2.0)	8 (5.4)	6 (4.0)
Left renal	33 (22.1)	8 (5.4)	2 (1.3)	3 (2.0)
Right renal	36 (24.2)	5 (3.4)	2 (1.3)	6 (4.0)
Superior mesenteric	15 (10.1)	12 (8.1)	1 (0.7)	6 (4.0)
Inferior mesenteric	6 (4.0)	3 (2.0)	0	0
**Pelvic**				
Left iliac	10 (6.7)	4 (3.4)	5 (3.4)	3 (3.0)
Right iliac	12 (8.1)	8 (5.4)	4 (2.7)	3 (2.0)
Superficial femoral	0	3 (2.0)	0	0
Deep femoral	0	1 (0.7)	0	0
**Others**	2 (1.3)	0	5 (3.4)	4 (2.7)

Data are expressed as frequency (%).

In addition, we conducted a comprehensive analysis of the association between the presence of upper or lower limb claudication and the initial clinical and radiological presentations previously detailed in Tables 1 , 2 , and 3 . The parameters demonstrating a statistical association are presented in Table 4 . For the upper limb, significant correlations were identified with dizziness, visual disturbances, diminished upper limb pulses, systemic arterial hypertension, left subclavian artery stenosis, right subclavian artery occlusion, and right subclavian artery thickening (all p<0.05). Regarding the lower limb, notable associations were observed with cardiac murmur, left subclavian artery stenosis, right subclavian artery occlusion, and right subclavian artery thickening (all p<0.05). In the multivariate analysis, the independent predictors for upper limb vascular claudication were reduced upper limb pulses (OR=4.83; 95%CI=2.08-11.24; p<0.001) and right subclavian artery occlusion (OR=8.06; 95%CI=1.94-33.44; p=0.004). For lower limb claudication, the primary predictors were left subclavian artery stenosis (OR=2.71; 95%CI=1.21-60.56; p=0.016), right subclavian artery occlusion (OR=6.65; 95%CI=2.05-21.61; p=0.002), and right subclavian artery thickening (OR=5.12; 95%CI=1.18-22.71; p=0.029).

**Table 4:** – Statistically significant associations between the presence of upper or lower limb claudication and initial clinical manifestations and radiological findings

	Vascular claudication		p value
Upper limbs	Present (n=54)	Absent (n=95)	
Dizziness	11 (20.4)	7 (7.4)	0.034
Visual disturbances	10 (18.5)	6 (6.3)	0.028
Reduced upper limb pulses	42 (77.8)	32 (33.7)	<0.001
Systemic arterial hypertension	16 (29.6)	48 (50.5)	0.016
L. subclavian a. stenosis	24 (44.4)	25 (26.3)	0.030
R. subclavian a. occlusion	13 (24.1)	3 (3.2)	<0.001
R. subclavian a. thickening	8 (14.8)	2 (2.1)	0.005
**Lower limbs**	**Present (n=46)**	**Absent (n=103)**	**p value**
Cardiac murmur	1 (2.2)	16 (15.5)	<0.001
L. subclavian a. stenosis	21 (45.7)	28 (27.2)	<0.001
R. subclavian a. occlusion	11 (23.9)	5 (4.9)	0.001
R. subclavian a. thickening	7 (15.2)	3 (2.9)	0.010

Data are expressed as frequency (%). R: right; L: left; a: artery.

Tables 5 and 6 provide a summary of the follow-up, treatment, and outcome data. The median follow-up period was 120 (49-178) months. At the final evaluation, over 90% of patients were in clinical remission. There was a significant reduction in the use of prednisolone (p<0.001) and immunosuppressive/immunomodulatory agents, such as methotrexate (p<0.001), azathioprine (p<0.001), and leflunomide (p<0.001).

**Table 5:** – Treatment and follow-up of patients with Takayasu arteritis

	Baseline	Final	p value
**Follow-up time (months)**	120 (49-178)		
**Treatment**			
Prednisolone	107 (71.8)	28 (18.8)	<0.001
**IS/IM/IB drugs**			
None	25 (16.8)	91 (61.1)	<0.001
Methotrexate	110 (73.8)	23 (15.4)	<0.001
Azathioprine	55 (36.9)	22 (14.8)	<0.001
Leflunomide	30 (20.1)	6 (4.0)	<0.001
Mycophenolate mofetil	26 (17.4)	8 (5.4)	<0.001
Infliximabe	25 (16.8)	15 (10.1)	0.009
Cyclophosphamide	12 (8.1)	1 (0.7)	0.003
Hydroxychloroquine	11 (7.4)	2 (1.3)	0.008
Tocilizumab	11 (7.4)	4 (2.7)	0.023
Adalimumab	4 (2.7)	1 (0.7)	0.248
Sulfasalazine	3 (2.0)	0	0.248
Abatacept	3 (2.0)	1 (0.7)	0.480
Etanercept	2 (1.3)	0	NA
Rituximab	1 (0.7)	0	NA
Colchicine	1 (0.7)	0	NA
Certolizumab pegol	1 (0.7)	0	NA
Tacrolimus	0	0	NA
Acetylsalicylic acid	-	119 (79.9)	NA
Statins	-	93 (62.4)	NA

Data are expressed as median (25th - 75th percentile) or frequency (%). P-values calculated using McNemar’s test. Baseline: at the time of TAK diagnosis and start of outpatient follow-up; Final: latest outpatient evaluation. IB: immunobiological; IM: immunomodulatory; IS: immunosuppressive. NA (Not Applicable): Statistical test not performed due to low frequency, missing data pairs, or because the medication was only assessed at one time point. The symbol “–” indicates data not systematically collected at the specified time point, whereas “0” indicates that the parameter was assessed and the recorded value was zero.

**Table 6:** – Clinical outcomes, complications, and mortality during follow-up

	Baseline	Final
**Disease in remission**	-	136 (91.3)
**Complications**		
Stroke	18 (12.1)	23 (15.4)
Congestive heart failure	11 (7.4)	22 (14.8)
Acute myocardial infarction	6 (4.0)	12 (15.4)
Systemic arterial hypertension	64 (43.0)	110 (73.8)
Aortic insufficiency	16 (10.7)	25 (16.6)
DVP/PE	-	7 (4.7)
Neoplasms	-	5 (3.4)
Dyslipidemia	-	84 (56.4)
Diabetes mellitus	-	18 (12.1)
**Vascular**		
Infarction/Ischemia of other organs	-	6 (4.0)
Aortic dissection	-	5 (3.4)
**Surgeries**		
Endoprosthesis	-	40 (26.8)
By-pass	-	9 (6.0)
Aortic valve replacement	-	9 (6.0)
Death	-	5 (3.4)

Data are expressed as median (25th - 75th), or frequency (%). Baseline: at the time of TAK diagnosis and start of outpatient follow-up; Final: latest outpatient evaluation. DVT: deep vein thrombosis; PE: pulmonary embolism. The symbol “–” indicates data not applicable or not systematically collected at the specified time point.

Overall mortality was recorded at 3.4%.

During the follow-up period, an increase in the prevalence of comorbidities, including systemic arterial hypertension, stroke, congestive heart failure, and acute myocardial infarction, was observed. Additionally, vascular complications such as infarction of other organs and aortic dissection were also noted ( Table 6 ).

Furthermore, there was an increase in the frequency of surgical procedures, including endoprosthesis implantation (26.8%), revascularization surgeries (6.0%), and aortic valve replacements (6.0%). The details are presented in Table 1 (Supplement) .

## Discussion

Our findings reveal that at the initial diagnosis of TAK, 7.4% of patients were asymptomatic. Among those presenting with symptoms, the clinical manifestations were predominantly nonspecific. Approximately half of the patients demonstrated significant vascular involvement, such as diminished pulses and a systolic blood pressure discrepancy in the upper limbs. Imaging revealed several alterations related to sequelae, including stenoses and occlusions, potentially indicative of the disease’s late diagnosis. The upper limb claudication was predicted by reduced upper limb pulses and right subclavian artery occlusion, whereas lower limb claudication was not predicted by lesions in infrarenal vessels but rather by severe involvement of the subclavian arteries.

The strengths of this study include a representative sample of patients with TAK, enrolled according to the most recent 2022 EULAR/ACR classification criteria.^22^Although this study was retrospective, data were systematically collected from electronic health records using pre-parameterized, standardized instruments, thereby minimizing measurement bias and ensuring data quality and uniformity.

Notably, unlike other studies in the literature,^6 , 8 - 12 , 14 - 20^our research specifically evaluated detailed initial manifestations and their correlation with prognosis, while also considering surgical events. For example, Alnabwani et al.^6^focused solely on stenoses without specifying imaging timing, whereas Gloor et al.^8^identified affected vessels without detailing anatomical alterations. Similarly, Cong et al.^9^and Vanoli et al.^10^either did not clearly define the imaging timeframe or failed to consistently differentiate between initial and late radiological findings.

Numerous studies^11 , 12 , 14 - 16^have failed to specify the timing of radiological changes, thus impeding precise correlation with initial symptoms. Even studies that considered early findings, such as that by Karabacak M et al.,^17^did not specify the type of radiological abnormalities detected. Furthermore, the research conducted by Thakare et al.^18^and Misra et al.,^19^while addressing initial aspects, limited their prognostic analyses to specific comparisons—renal involvement or pulse status, respectively—presenting data in ratio formats, which restricts the comprehensive correlation of initial clinical and radiological features with overall prognosis. Notably, unlike our study, these works did not detail surgical events. Additionally, the study by Uchida et al.,^20^despite providing initial and prognostic data, was constrained by a short follow-up period of only two years and also omitted reports of surgical interventions.

Our findings revealed that at the time of TAK diagnosis, 92.6% of patients were symptomatic. However, they exhibited varied and highly nonspecific clinical symptoms, such as fever and weight loss. In contrast, typical symptoms like vascular claudication of the upper and lower limbs, although more specific, were present in less than half of the patients. The upper limb claudication was exclusively associated with diminished upper limb pulses and right subclavian artery occlusion, despite its initial correlation with parameters such as dizziness and visual disturbances. This finding suggests that upper limb claudication serves as a significant clinical marker of advanced vascular disease, correlating with indicators of established vascular damage and underscoring the late diagnosis of the condition. Our results further indicate that claudication does not necessarily reflect initial clinical manifestations but rather specific vascular alterations, emphasizing the importance of physical examination and radiological assessment in patient evaluation.

The lower limb claudication was initially associated with cardiac murmur and various radiological alterations; multivariate analysis revealed no association with infrarenal aortic vessel lesions or initial symptoms. Instead, it was linked to vascular lesions in suprarenal territories, with independent predictors including left subclavian artery stenosis, right subclavian artery occlusion, and right subclavian artery thickening. This unexpected correlation suggests that lower claudication may not reflect localized arterial involvement but rather indicates severe subclavian artery involvement as a marker of disease stage, reflecting significant vascular compromise with global hemodynamic repercussions. As with upper claudication, these findings support the hypothesis of a late diagnosis, occurring when vascular damage is already functionally significant, highlighting the importance of imaging studies in patient management.

Additionally, the prevalence of hemodynamic manifestations in approximately half of the patients at diagnosis, along with vascular alterations such as stenoses and occlusions in the majority, provides evidence for the late diagnosis of TAK.

The statistical analysis concentrated on vascular claudication symptoms due to their clinical significance and prevalence within our cohort. While systemic manifestations such as fever, fatigue, and headache were prevalent at diagnosis, their nonspecific nature limits the ability to establish direct and robust correlations with specific radiological findings. In contrast, more specific symptoms were rare, precluding a statistical analysis with sufficient power to yield valid conclusions. Consequently, the analysis was conducted based on upper and lower limb claudication to correlate initial clinical manifestations and imaging findings in TAK.

Our findings further revealed that although a substantial majority of patients achieved remission, there was a notable increase in cardiovascular comorbidities and the necessity for complex surgical interventions. This indicates that clinical remission does not invariably equate to anatomical stability or the reversal of established vascular damage, highlighting a critical dissociation between the management of inflammatory activity and the long-term progression of vascular damage.

The severity and advancement of the disease are underscored by the high incidence of surgical interventions, such as the implantation of endoprostheses in over a quarter of the patients, alongside revascularization surgeries and aortic valve replacements. The occurrence of severe vascular complications, such as aortic dissection during follow-up, further accentuates this progression, indicating that the disease can advance with extremely severe vascular events. This is evidenced by the causes of death, which included events such as extensive ischemic stroke and septic shock. These data underscore that, even with the control of inflammation, the accumulated vascular damage continues to result in significant morbidity, necessitating rigorous and continuous monitoring.

This study has some methodological limitations. First, its retrospective design may introduce information bias and depends on the accuracy and completeness of electronic medical records. Second, being a single-center study, the findings may lack generalizability to broader populations with different ethnic or genetic backgrounds. These limitations underscore the need for future prospective, multicenter studies to validate and expand upon our results.

## Conclusions

This study elucidates that TAK is characterized by nonspecific symptoms that coincide with advanced vascular lesions, such as stenoses and occlusions. The direct correlation between specific clinical manifestations, such as claudication, and these lesions underscores the prognostic significance of the initial findings. Over the long term, despite a high rate of clinical remission, the disease exhibited progression, marked by an increase in cardiovascular comorbidities and the necessity for surgical interventions, thereby addressing a gap in the literature concerning the progression of TAK. Prospective, multicenter studies are warranted to validate these findings.

## Supplemental Materials

Table 1 (Supplement)
